# Routine treatment and outcome of breast cancer in younger versus elderly patients: results from the SENORA project of the prospective German TMK cohort study

**DOI:** 10.1007/s10549-017-4534-8

**Published:** 2017-10-13

**Authors:** Thomas Fietz, Mark-Oliver Zahn, Andreas Köhler, Erik Engel, Melanie Frank, Lisa Kruggel, Martina Jänicke, Norbert Marschner, the TMK-Group (Tumour Registry Breast Cancer)

**Affiliations:** 1 Outpatient-Centre for Internal Medicine, Haematology and Oncology, Virchowstr. 10c, 78224 Singen, Germany; 2 Ambulatory Healthcare Centre (MVZ) Oncological Cooperation Harz, Kösliner Str. 14, 38642 Goslar, Germany; 3 Outpatient-Centre for Haematology and Oncology, Röntgenstr. 6-8, 63225 Langen, Germany; 4 Haematological-Oncological Practice Altona (HOPA), Hamburg, Germany; 5 Statistics, iOMEDICO, Hanferstr. 28, 79108 Freiburg, Germany; 6 Clinical Epidemiology and Health Economics, iOMEDICO, Hanferstr. 28, 79108 Freiburg, Germany; 7 Outpatient-Centre for Interdisciplinary Oncology and Haematology, Wirthstrasse 11c, 79110 Freiburg, Germany

**Keywords:** Breast neoplasms, Aged, Registries, Cohort studies, Outcome assessment, Prognosis

## Abstract

**Purpose:**

There is an ongoing discussion about ‘undertreatment’ of breast cancer in elderly patients. Due to low accrual into clinical trials, level 1 evidence is scarce. We report prospective data of elderly patients with breast cancer treated by medical oncologists in Germany.

**Methods:**

The SENORA project within the prospective cohort study TMK (Tumour Registry Breast Cancer) was conducted in 82 centres from 2007–2015. Among 2316 patients, half were enrolled with curative and half with palliative treatment intention. Overall, 478 patients (21%) were aged ≥ 70.

**Results:**

In the adjuvant setting, elderly patients aged ≥ 70 had more advanced tumour stages at diagnosis and a higher prevalence of comorbidities than younger patients. Elderly patients received adjuvant chemotherapy less frequently, yet the 3-year disease-free survival was similar (86% vs. 88%). In the palliative setting, elderly patients more frequently received endocrine therapy and less frequently chemotherapy. Their median overall survival [24.9 months, 95% CI (confidence interval) 20.0–30.2] was significantly shorter than that of younger patients (39.7 months, 95% CI 34.9–44.2). A Cox proportional hazards model showed a significantly increased risk of mortality for: age ≥ 70 at start of therapy, negative HR- or HER2-status, higher number of metastatic sites, more comorbidities and high tumour grading at diagnosis.

**Conclusions:**

Our results shed light on the routine treatment of elderly patients with breast cancer. A regression model demonstrated that age is but one of various prognostic factors determining the shorter overall survival of elderly patients.

## Introduction

In Germany, breast cancer is the most frequently diagnosed cancer (31% of all cases), with women aged 70 or older at diagnosis representing 34% of these cases [1, 2]. In general, the risk of being diagnosed with breast cancer increases with age, affecting 1 in 48 women at age 45 and 1 in 28 women at age 65 [1]. The number of elderly individuals in our population is growing rapidly. While 17% of the German population was aged 60 and older in 1960, this proportion increased to 27% in 2013 [3]. Thus, the number of elderly patients with breast cancer continues to rise. Since elderly patients are more often affected by comorbidities or have an inferior general condition, the therapeutic options might need to be adjusted. As a woman aged 70 has a life expectancy of 16.8 years in Germany [4], treatment decision should not be guided by the chronological age, but needs to balance the risks and benefits, health status, life expectancy, individual preferences and sustained quality of life.

Prognostic factors influencing breast cancer outcome relate to tumour and patient characteristics [5–7]; the interactions between these factors are complex and not yet fully understood. In comparison to younger patients, breast tumours in older women tend to have less aggressive profiles and lower recurrence rates [8, 9], but the relative survival in women older than 70 has been reported to be lower [5, 10, 11]. Hampered by the underrepresentation of women aged ≥ 70 in clinical trials, there are only limited data on treatment of this group of patients [12–15]. Several reports have stated deviations from the guidelines in treatment of breast cancer in elderly patients, especially concerning surgery and adjuvant chemotherapy [16–19]. This so-called undertreatment might be due to missing clinical evidence as the standard treatment is based on recommendations extrapolated from younger patients participating in clinical trials [14, 20]. In addition, when assessing guideline conformity, factors preventing use of recommended treatments (such as comorbidities) are often missing. There is an urgent need for prospective clinical trials as well as population-based cohort studies tailored for elderly patients in order to optimise their treatment.

In this article, we present data on 2316 patients with breast cancer from the SENORA project within the TMK (Tumour Registry Breast Cancer). This prospective clinical cohort study recruits patients treated by office- and clinic-based medical oncologists, thus showing data from routine practice. We show patient and tumour characteristics as well as treatment details for the adjuvant and the palliative setting, both for younger patients and for patients aged ≥ 70. Furthermore, we present the disease-free survival (DFS) as well as the overall survival (OS) for both younger and elderly patients and additionally present a multivariate regression model identifying factors influencing the OS.

## Patients and methods

### Data source

The TMK is an ongoing, open, longitudinal, multicentre, observational, prospective cohort study which started in 2007. The study was approved by the responsible ethics committee and is registered at ClinicalTrials.gov (NCT01351584). Eligible patients are women aged ≥ 18 years with histologically confirmed breast cancer, who received systemic antineoplastic treatment (endocrine therapy or chemotherapy). Written informed consent was obtained from all patients. Enrolment was restricted to patients who had signed informed consent no longer than 6 weeks after start of treatment, yet for the first 1000 patients with palliative treatment intention, the maximum span between start of first-line treatment and enrolment was 1 year. By project plan, patients were followed until death or for a maximum of 3 years. However, some sites provided update data on survival status after completion of the 3 year follow-up. The SENORA sub-project was set up in order to examine the treatment of elderly patients with breast cancer. 82 outpatient-centres and clinics for medical oncology located all over Germany actively recruited patients for the SENORA project between 2007 and 2011. The TMK has previously been described in detail [21].

### Cohort definition

**Fig. 1:**
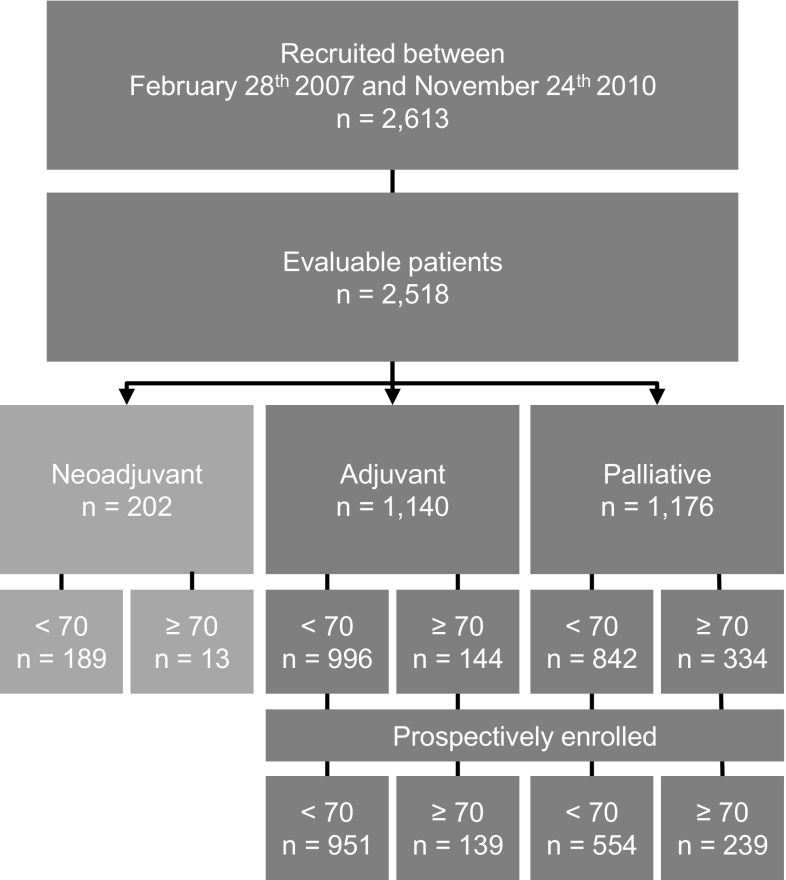
Cohort definition. Number of patients enrolled in the TMK, split up according to the neoadjuvant, adjuvant or palliative treatment intention as well as to the age at start of therapy. Prospectively enrolled patients signed the informed consent no longer than 6 weeks after start of treatment

Between 2007 and 2011, 2613 patients had been recruited into the TMK. 74 patients were excluded because of incomplete basic medical data or not fulfilling inclusion criteria. 21 patients who received trastuzumab monotherapy were excluded as the sample size was too small for a separate group. Of the remaining 2518 patients, 202 received neoadjuvant therapy but only 13 of them were aged 70 or older, therefore, this patient subgroup will not be discussed further. The present analysis focuses on 2316 patients receiving adjuvant or palliative first-line chemo- or endocrine therapy (Fig. 1). 1838 of the patients were younger than 70, 478 patients were aged ≥ 70. Outcome data including the OS are presented only for the prospectively enrolled patients, who had signed informed consent no longer than 6 weeks after the start of treatment to avoid overestimation of outcome (Fig. 1).

### Statistical analysis

The aim of the SENORA project was the descriptive comparison of patient characteristics, treatment and outcome of elderly (age ≥ 70) versus younger patients. Time to events was analysed using Kaplan–Meier estimates. For the prospectively enrolled patients in adjuvant treatment intention, DFS was defined as the interval between start of adjuvant treatment and the date of recurrence or death from any cause. Patients lost to follow-up or without recurrence were censored at last contact. For the prospectively enrolled patients with palliative treatment intention, PFS was defined as the interval between start of first-line therapy and the date of first progression or death. Patients without such an event before start of second-line therapy were censored at either the start of second-line therapy or at last contact, whatever came first. OS was defined as the interval between start of first-line therapy and the date of death from any cause. Patients alive or lost to follow-up were censored at last contact. DSS was calculated from start of first-line therapy until date of death due to breast cancer. Patients alive, lost to follow-up or with other causes of death were censored at last contact or at date of death. The treatment duration was calculated using a Kaplan–Meier estimate and defined as time from start of treatment until end of treatment or death. If no end of therapy was documented, patients were censored at last contact/end of documentation. The median follow-up period was calculated using the reverse Kaplan–Meier estimate [22]. All analyses were performed using IBM SPSS Statistics version 19.0 and SAS for Windows version 9.4. Exact binomial confidence intervals were calculated using *z*-test with StatSoft STATISTICA version 13.

For the prospectively enrolled patients with palliative treatment intention (maximum span of 6 weeks between start of treatment and signed informed consent), a Cox proportional hazards model was used to identify potential independent prognostic factors for survival. The following independent variables were examined for the model: age at start of first-line treatment, body mass index (BMI) at enrolment, Charlson comorbidity index, CCI [23] at diagnosis, HR-status and HER2-status at diagnosis, stage and tumour grading at diagnosis as well as resection of the primary tumour. Furthermore, these treatment details at start of first-line therapy were included: prior neoadjuvant or adjuvant chemotherapy; number of metastatic sites and location of metastases. Nonvisceral metastases were defined as skin, bone or lymph node metastases. Confidence intervals (CI) for the regression coefficients were based on the Wald statistics. All presented *P* values are two-sided. 5% will be interpreted as significant. There were no adjustments to the level of significance.

## Results

### Patient and tumour characteristics in the adjuvant setting

Basic demographic and clinical data of the patients receiving adjuvant therapy are presented in Table 1, comparing patients younger than 70 years to those aged ≥ 70. Median age at start of adjuvant therapy was 55 years (< 70) compared to 73 years (≥ 70). The prevalence of comorbidities was higher for elderly (38% CCI ≥ 1) than for younger patients (18% CCI ≥ 1), with hypertension documented most frequently (57%). 35 (24%) of the elderly patients were ≥ 75 years old, 8 (6%) of them ≥ 80 years old. Regarding the receptor status, the two subgroups were comparable. Elderly patients more often presented with advanced tumour stage at diagnosis (26% stage III vs. 14% of the younger patients) and more often had lymph node involvement (58% vs. 48%).

**Table 1:** Patient and tumour characteristics in the adjuvant setting

Characteristic	< 70 (*n* = 996)		≥ 70 (*n* = 144)	
	Median	Min–Max	Median	Min–Max
Age at start of therapy, years	55.1	21.6–69.9	72.8	70.0–85.5

Characteristic	*n*	%	*n*	%
BMI at enrolment^a^				
Underweight	5	0.5	1	0.7
Normal	454	45.6	41	28.5
Overweight	294	29.5	53	36.8
Obese	223	22.4	44	30.6
Unknown	20	2.0	5	3.5
Patients with any comorbidity^b,c,e^	470	47.2	118	81.9
Hypertension	240	24.1	82	56.9
Diabetes mellitus	69	6.9	2	14.6
Cardiovascular disease	34	3.4	18	12.5
Charlson comorbidity index^d^				
CCI = 0	814	81.7	89	61.8
CCI ≥ 1	182	18.3	55	38.2
Receptor status at diagnosis				
HR-positive, HER2-negative	569	57.1	82	56.9
HR-positive, HER2-positive	176	17.7	19	13.2
HR-negative, HER2-positive	77	7.7	10	6.9
Triple negative	147	14.8	26	18.1
Unknown	27	2.7	7	4.9
Tumour stage at diagnosis				
I	268	26.9	26	18.1
II	443	44.5	60	41.7
III	141	14.2	38	26.4
Unknown^f^	144	14.5	20	13.9
Tumour grading at diagnosis				
G1	74	7.4	11	7.6
G2	540	54.2	64	44.4
G3	371	37.2	65	45.1
GX	11	1.1	4	2.8
Node stage at diagnosis				
N0	502	50.4	58	40.3
N1	341	34.2	47	32.6
N2	84	8.4	22	15.3
N3	56	5.6	15	10.4
Unknown	13	1.3	2	1.4

*BMI* body mass index (kg/m^2^), *HR* hormone receptor, *HER2* human epidermal growth factor receptor 2, *Max* maximum, *Min* minimum

^a^Underweight: BMI < 18.5; normal: BMI 18.5 to < 25, overweight: BMI 25 to < 30, obese: BMI ≥ 30

^b^At start of therapy

^c^At least one comorbidity according to Charlson [23] or additional concomitant diseases

^d^Charlson Comorbidity Index (CCI) according to Quan et al. [24]

^e^Multiple answers provided

^f^For some patients the exact stage could not be determined because of unknown parameters (TX, NX, MX)

### Patient and tumour characteristics in the palliative setting

Table 2 presents the basic characteristics of the two patient subgroups receiving palliative therapy. Median age at diagnosis was 53 versus 72 years, the BMI was similar. 75% of the elderly patients presented with comorbidities compared to 49% of the younger patients. 169 (51%) of the elderly patients were ≥ 75 years old, 66 (20%) of them ≥ 80 years old. 49% of the patients aged ≥ 70 had hypertension (vs. 29% of the younger patients) and 20% diabetes mellitus (vs. 7%). Generally, the prevalence of comorbidities was higher in elderly patients in palliative therapy (38% CCI ≥ 1 vs. 18% CCI ≥ 1). The distribution of receptor subtypes was comparable in younger and older patients. Independent of age, a similar number of patients presented with synchronous metastasis (30 vs. 29%), with nonvisceral (bone, skin and/or lymph node) metastases (36 vs. 37%) or with more than one metastatic site (42 vs. 40%). Referring to the location of the metastases, slightly more of the younger patients had brain metastases (3 vs. 1%), whereas slightly more elderly patients had metastases in the lung (7 vs. 10%). The proportion of patients with preceding adjuvant chemotherapy was a bit higher in the younger patient group (58% vs. 51%).

**Table 2:** Patient and tumour characteristics in the palliative setting

Characteristic	< 70 (*n* = 842)		≥ 70 (*n* = 334)	
	Median	Min–Max	Median	Min–Max
Age at diagnosis, years	53.2	22.3–69.6	71.6	37.1–90.2
Age at start of therapy, years	59.1	22.5–70.0	75.1	70.0–92.8

Characteristic	*n*	%	*n*	%
BMI at enrolment^a^				
Underweight	7	0.8	6	1.8
Normal	344	40.9	123	36.8
Overweight	262	31.1	115	34.4
Obese	173	20.5	60	18.0
Unknown	56	6.7	30	9.0
Patients with comorbidity^d,e^	415	49.3	250	74.9
Hypertension	241	28.6	165	49.4
Diabetes mellitus	55	6.5	65	19.5
Cardiovascular disease	20	2.4	36	10.8
CCI = 0^f^	688	81.7	192	57.5
CCI ≥ 1	154	18.3	142	42.5
Receptor status at diagnosis				
HR-positive, HER2-negative	389	46.2	159	47.6
HR-positive, HER2-positive	162	19.2	54	16.2
HR-negative, HER2-positive	78	9.3	21	6.3
Triple negative	93	11.0	28	8.4
Unknown	120	14.3	72	21.6
Tumour grading at diagnosis				
G1/2	428	50.8	183	54.8
G3/4	314	37.3	115	34.4
GX/Unknown	100	11.9	36	10.8
Resection of primary tumour				
R0	636	75.5	250	74.9
R1/2	63	7.5	35	10.5
RX/unknown	143	17.0	49	14.7
Metastasis at diagnosis				
No (metachronous, M0)	489	58.1	184	55.1
Yes (synchronous, M1)	248	29.5	97	29.0
MX^b^/Unknown	105	12.5	53	15.9
Location of metastases^d,g^				
Visceral ± nonvisceral	494	58.7	191	57.2
Nonvisceral only	302	35.9	123	36.8
Unknown	46	5.5	20	6.0
Bone only	207	24.6	86	25.7
Liver only	76	9.0	33	9.9
Lung only	56	6.7	32	9.6
Brain	27	3.2	3	0.9
Multiple and/or other location^c^	430	51.1	160	47.9
Unknown	46	5.5	20	6.0
Number of metastatic sites^d^				
= 1	445	52.9	179	53.6
> 1	351	41.7	135	40.4
Unknown	46	5.4	20	6.0
Preceding adjuvant therapy	490	58.2	170	50.9

*BMI* body mass index (kg/m^2^), *HR* hormone receptor, *HER2* human epidermal growth factor receptor 2, *Max* maximum, *Min* minimum

^a^Underweight: BMI < 18.5; normal: BMI 18.5 to < 25, overweight: BMI 25 to < 30, obese: BMI ≥ 30

^b^MX, presence of distant metastasis was not evaluated or is not documented for the time of primary diagnosis

^c^Other locations than the aforementioned or multiple locations

^d^at start of palliative first-line therapy

^e^At least one comorbidity according to Charlson [23] or additional concomitant diseases

^f^Charlson Comorbidity Index (CCI) according to Quan et al. [24]

^g^Nonvisceral: bone, lymph node and/or skin metastases

### Treatment

**Fig. 2:**
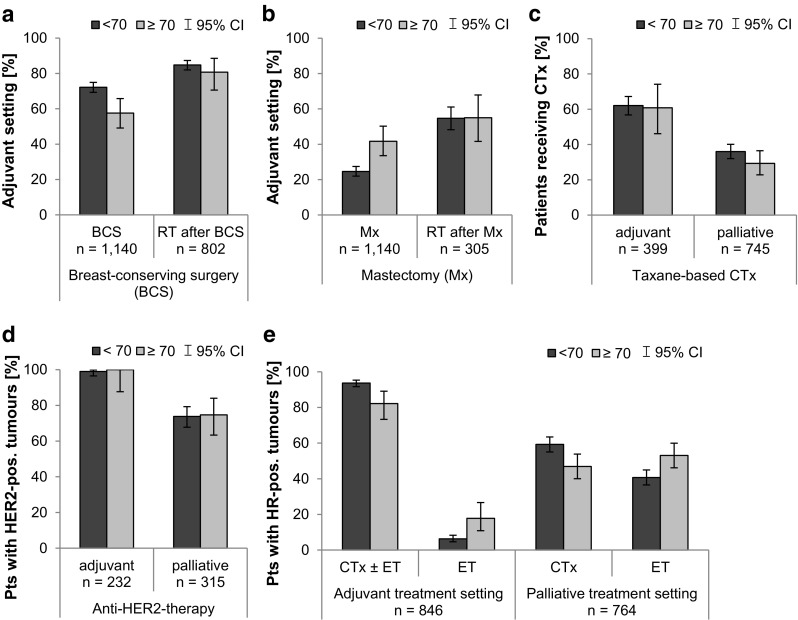
Treatment of breast cancer for patients aged < 70 in comparison to patients aged ≥ 70. **a** Proportion of patients receiving breast conserving surgery (BCS) in the adjuvant treatment setting. The proportion of patients receiving radiotherapy (RT) is calculated for all patients receiving BCS. **b** Proportion of patients receiving mastectomy (Mx) in the adjuvant treatment setting. The proportion of patients receiving radiotherapy is calculated for all patients receiving Mx. **c** Proportion of patients receiving taxane-based chemotherapy (of all patients receiving chemotherapy). **d** Proportion of patients receiving anti-HER2-therapy (of all patients with HER2-positive tumours). **e** Proportion of patients with HR-positive tumours receiving systemic chemotherapy and/or endocrine therapy. In the adjuvant treatment setting, patients receiving chemotherapy and endocrine therapy as well as patients receiving only chemotherapy were added up. 114 younger patients (15.3%) and 12 elderly patients (11.9%) received chemotherapy without endocrine therapy. Error bars represent the 95% confidence interval (CI) Abbreviations: *BCS* breast conserving surgery, *CI* confidence interval, *CTx* chemotherapy, *ET* endocrine therapy, *HER2* human epidermal growth factor receptor 2, *HR* hormone receptor, *Mx* mastectomy, *pos* positive, *pts* patients, *RT* radiotherapy

Patients aged ≥ 70 at start of therapy less often underwent a breast conserving surgery (BCS) than younger patients (58%, 95% CI 49–66% vs. 72%, 95% CI 69–75%, Fig. 2a), and more often had a mastectomy (42%, 95% CI 34–50% vs. 25%, 95% CI 22–27%, Fig. 2b). After surgery, however, the same proportion of patients in both subgroups received radiotherapy, 81–85% after BCS and 55% after mastectomy (Fig. 2a, b). The proportion of taxane-based chemotherapy was similar independent of the age in the adjuvant setting (61 vs. 62%), while fewer elderly patients received this regimen in palliative first-line (29%, 95% CI 23–37% vs. 36%, 95% CI 32–40%, Fig. 2c). For patients with HER2-positive tumours, administration of anti-HER2-therapy in the adjuvant and palliative first-line setting was similar between the age groups (Fig. 2d). A higher proportion of elderly patients with HR-positive tumours received endocrine therapy only, 18% (95% CI 11–27%) versus 6% (95% CI 5–8%) in the adjuvant and 53% (95% CI 46–60%) versus 41% (95% CI 37–45%) in the palliative setting (Fig. 2e).

### Treatment response and outcome data

For all prospectively enrolled patients (see Fig. 1), response rates and outcome data according to the different treatment regimens are shown in Table 3. The median follow-up period was 41.3 months (range 33–53 months) for all patients, with only a slight difference between the age groups: 42.8 months (range 33–54 months) vs. 37.9 months (range 29–47 months) for patients aged ≥ 70.

**Table 3:** Outcome data in the adjuvant and palliative setting

Adjuvant setting (prospectively enrolled patients)	< 70 (*n* = 951)		≥ 70 (*n* = 139)	
Patients receiving chemotherapy (*n*, %)	714	75.1%	92	66.2%
Number of cycles (median, min–max)^a^	6.0	3.0–6.0	4.0	3.0–6.0
Duration of CTx in months (median, 95% CI)^b^	3.5	NA–NA	3.5	3.5–3.6
End of CTx documented^c^ (*n*, %)	709	99.3%	91	98.9%
Patients receiving targeted therapy (*n*, %)	192	20.2%	28	20.1%
Number of cycles (median, range)^d^	17.0	13.0–18.0	18	12.0–18.0
Duration of TT in months (median, 95% CI)	14.7	14.2–15.6	15.2	11.7–16.6
End of TT documented^c^ (*n*, %)	189	98.4%	27	96.4%
Patients receiving endocrine therapy (*n*, %)	615	64.7%	90	64.7%
Duration of ET in months (median, 95% CI)	59.5	58.5–61.9	59.2	43.2–NA
End of ET documented^c^ (*n*, %)	159	25.9%	25	27.8%
Disease-free survival—all patients and treatments	951	100%	139	100%
Events (*n*, %)	110	11.6%	19	13.7%
Median DFS	NA		NA	
Survival rate (%, 95% CI)				
12 months	96.3%	94.8–97.3	94.0%	88.3–97.0
24 months	91.4%	89.2–93.1	90.2%	83.4–94.4
36 months	87.8%	85.3–89.9	85.5%	77.1–91.0

Palliative first-line setting (prospectively enrolled patients)	< 70 (*n* = 554)		≥ 70 (*n* = 239)	
Patients receiving endocrine therapy (*n*, %)	132	23.8%	85	35.6%
Duration of ET in months (median, 95% CI)	15.9	13.3–20.2	15.4	10.9–20.1
Best response				
CR/PR	26	19.7%	12	14.1%
SD	46	34.8%	27	31.8%
PD	24	18.2%	19	22.4%
Missing	36	27.3%	27	31.8%
Patients receiving chemotherapy (*n*, %)	422	76.2%	154	64.4%
Duration of CTx^a^ in months (median, 95% CI)	5.1	4.6–5.8	3.8	3.5–4.9
Best response				
CR/PR	173	41.0%	52	33.8%
SD	115	27.3%	41	26.6%
PD	66	15.6%	34	22.1%
Missing	68	16.1%	27	17.5%
Progression-free survival	420	75.8%	152	63.6%
Events (*n*, %)	237	56.2%	102	66.2%
Median PFS (months, 95% CI)	12.2	10.8–13.3	7.6	6.6–8.9
Overall survival—all patients and treatments	554	100%	239	100%
Events (*n*, %)	275	49.6%	146	61.1%
Median OS (months, 95% CI)	39.7	34.9–44.2	24.9	19.9–30.1
Survival rate (%, 95% CI)				
12 months	83.8%	80.4–86.7	71.6%	65.3–77.0
24 months	68.2%	63.9–72.1	51.6%	44.7–58.0
36 months	53.5%	48.8–57.9	37.8%	31.0–44.5

Duration of therapy was calculated with a Kaplan–Meier estimate

*CI* confidence interval, *CR* complete response, *CTx* chemotherapy, *ET* endocrine therapy; NA, not available (not reached); OS, overall survival, *PD* progressive disease, *PFS* progression-free survival, *PR* partial response, *SD* stable disease, *StD* standard deviation, *TT* targeted therapy

^a^For all patients with available data on this parameter: < 70, *n* = 708; ≥ 70, *n* = 91

^b^Without targeted therapy (TT)

^c^Patients who completed the respective treatment within the follow-up period of this cohort study. Percentages refer to all patients receiving the respective treatment

^d^For all patients with available data on this parameter: < 70, *n* = 179; ≥ 70, *n* = 25

**Fig. 3:**
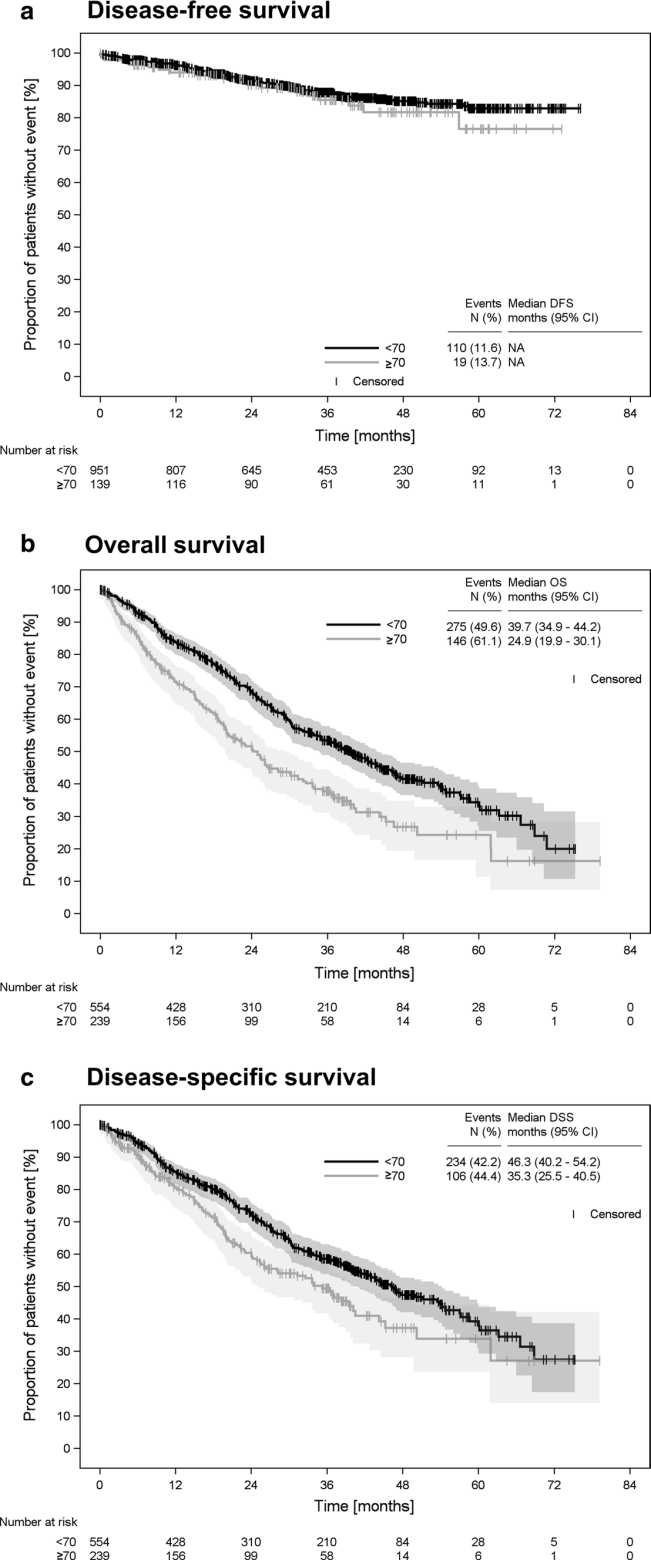
Survival of patients aged < 70 compared to patients aged ≥ 70. Survival analysis for the prospectively enrolled patients (see Fig. 1). **a** DFS for the patients with (neo)adjuvant treatment intention, **b** OS for the patients with palliative treatment intention, **c** Disease-specific survival for the patients in the palliative setting. Abbreviations: *CCI* Charlson Comorbidity Index, *CI* confidence interval, *DFS* disease-free survival; *DSS* disease-specific survival, *OS* overall survival

In the adjuvant setting, duration of chemotherapy did not differ between younger and elderly patients. The DFS rates were comparable: 88% of the younger patients lived at least 36 months without recurrence compared to 86% of the patients aged ≥ 70 (Table 3, Fig. 3a).

In the palliative first-line setting, duration of endocrine therapy in patients with HR-positive tumours was comparable (15.9 months, 95% CI 13.3–20.2 vs. 15.4 months, 95% CI 10.9–20.1). Endocrine therapy was successful in 55% younger and 46% elderly patients, as assessed by the disease control rate (DCR), covering complete/partial response and stable disease (CR/PR and SD; Table 3). Chemotherapy was successful in 68% of the younger and 60% of the elderly patients (DCR, Table 3). The median PFS was 12.2 months (95% CI 10.8–13.3) for the younger and 7.6 months (95% CI 6.6–8.9) for the elderly patients treated with chemotherapy. The OS analysis, calculated from start of first-line therapy, revealed that patients aged ≥ 70 had a significantly shorter median OS of 24.9 months (95% CI 20.0–30.2) compared to 39.7 months (95% CI 34.9–44.2) for younger patients (Fig. 3b). Breast cancer was documented as cause of death for 85% of the younger patients and for 73% of the patients aged ≥ 70. Median disease-specific survival was 46.3 months (95% CI 40.2–54.2) for younger and 35.3 (95% CI 25.5–40.5) months for elderly patients (Fig. 3c).

### Factors influencing overall survival

**Fig. 4:**
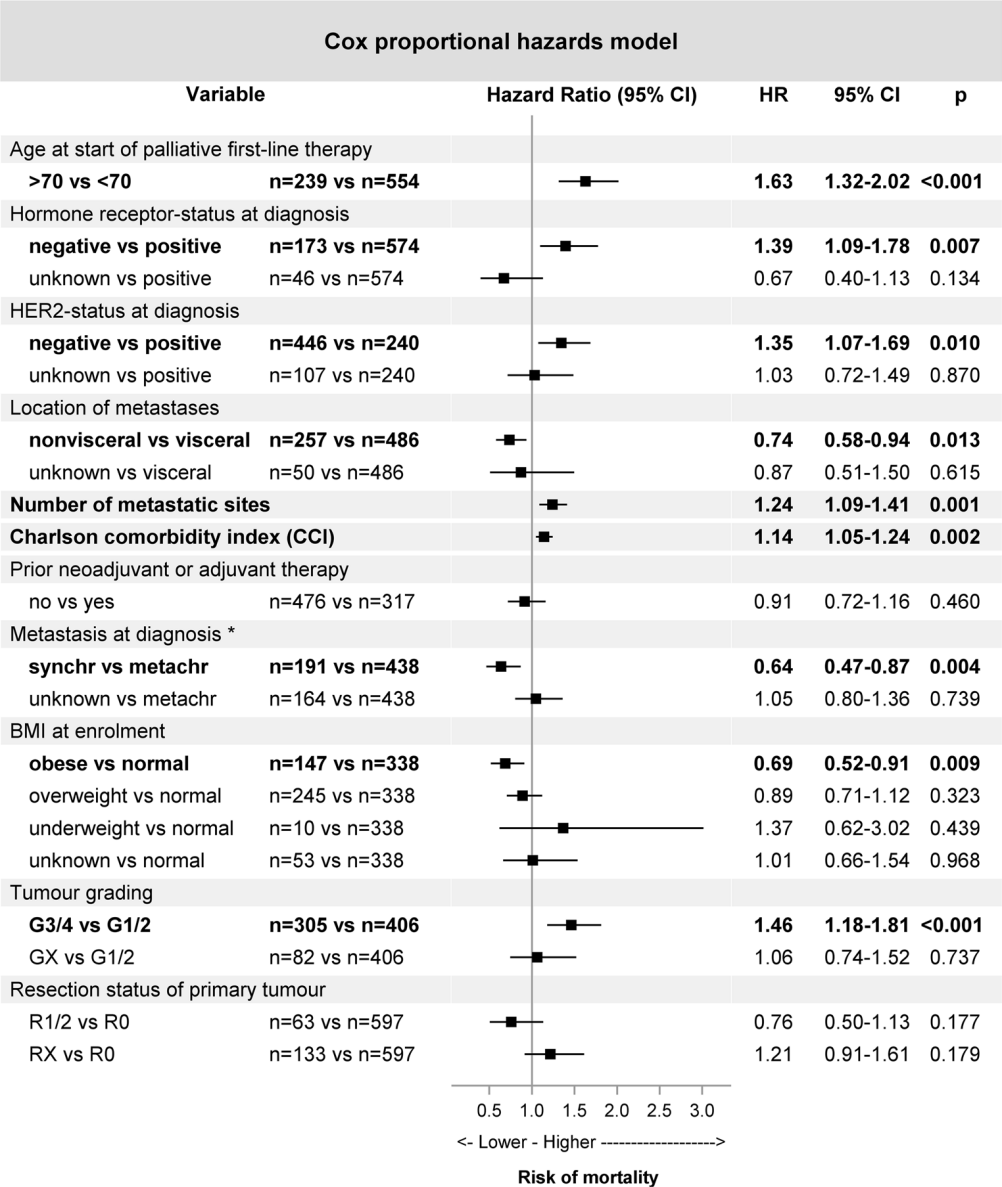
Multivariate regression analysis. Cox proportional hazards model for overall survival. Bold writing: significant results (*p* < 0.05). *Metastasis at diagnosis was documented either synchronous (M1) or metachronous (M0). Abbreviations: *BMI* body mass index, *CCI* Charlson Comorbidity Index, *CI* confidence interval, *HR* hazard ratio, *HER2* human epidermal growth factor receptor 2, *metachr* metachronous, *synchr* synchronous

A multivariate regression analysis showed that not only an age of ≥ 70 at start of palliative first-line therapy, but also negative HR- and HER2-status, higher number of metastatic sites, higher CCI and high tumour grading at diagnosis were associated with a significantly increased risk of overall mortality (Fig. 4). In contrast, nonvisceral metastases (vs. visceral metastases), metastasis at diagnosis (synchronous (M1) vs. metachronous (M0)) as well as obesity (obese vs. normal) were associated with a decreased risk of mortality (Fig. 4).

## Discussion

The proportion of elderly patients with breast cancer is growing continuously. Treatment decisions should not be based on chronological age alone, but need to ensure that elderly patients get the best quality of care. We shed light on the medical management of patients aged ≥ 70 in routine care, presenting data on treatment and outcome of 478 elderly and 1838 younger patients. In the adjuvant setting, elderly patients less often received chemotherapy. The 3-year DFS was similar to that of younger patients. In the palliative setting, the median OS of elderly patients was significantly shorter than that of younger patients, despite similar treatment. However, not only the age at start of therapy, but also the presence of comorbidities or metastases, negative HR- or HER2-status or high tumour grading at diagnosis significantly increased the risk of mortality.

This study is limited by the sole enrolment of patients receiving systemic therapy, potentially excluding patients refusing such treatment as well as very frail older patients not able to receive systemic therapy. The interpretation of data from cohort studies is limited by the lack of randomization which has to be considered when comparing outcome data between subgroups. In the TMK registry, there are no specifications as to the timing, frequency or criteria of tumour assessment and, thus DFS and PFS data should be considered as the best clinical approximation and might not be identical to the PFS determined in clinical trials. Strengths of this study are the prospective, longitudinal design and the participation of oncologists all over Germany.

At what chronological age is a patient defined as ‘elderly’? Even this simple question raises multiple issues and complicates comparisons between studies. Some authors set the threshold at ≥ 65 years [13], others at ≥ 70 [10, 11, 25] or even at ≥ 80 years [26]. The NCCN task force report on breast cancer in the older woman recommended the cutoff point ‘70 years or older’, as there is almost no clinical evidence for treatment of this patient subgroup [14], rendering prospective cohort studies like ours important information resources on the patients aged ≥ 70.

Poor diagnostic assessment, one of the factors for substandard treatment, was not observed in the SENORA cohort. The proportion of adjuvant patients with unknown/undocumented receptor status, tumour or nodal stage was similar in both age groups. It has been shown that patients aged ≥ 55 often have a more advanced disease at diagnosis, yet slower proliferation rates and less aggressive tumour profiles, including a higher proportion of HR-positive tumours [8]. Consistent with these reports, 26% of the elderly patients in the SENORA cohort were initially diagnosed with tumour stage III compared to 14% of the younger patients. The finding that patients aged > 70 more frequently present with more advanced tumours might be a consequence of recommendations for screening mammograms, which have only been established for the age groups between 50 and 70 years [27]. The HR-positive tumours, however, were equally distributed over the age groups. Although no differences in tolerance of BCS or mastectomy have been shown due to age [28], elderly patients are more often treated with mastectomy [13]. Indeed, our results show that 42% of the elderly patients underwent mastectomy, compared to 24% of the younger women. This tendency is in concordance with the NORA cohort study showing that 49% of the patients aged ≥ 70 versus 40% of the patients aged 65–69 were treated with mastectomy [13]. The value of adjuvant chemotherapy in the elderly population is controversial (reviewed in 30) and it has been reported that few elderly patients who are candidates for chemotherapy actually receive it [19, 29]. Of the prospectively enrolled patients with adjuvant treatment intention, 75% of the younger and 66% of the elderly patients received adjuvant chemotherapy, although the proportion of patients with more advanced tumour stages was higher in the elderly group. Interestingly, the 3-year DFS rate was similar between both groups and comparable to previously published data [30]. The number of cycles and duration of treatment was similar between the age groups. In short, ‘substandard treatment’ of elderly patients was not observed in our setting.

For the patients aged ≥ 70 with palliative treatment intention, median OS was significantly shorter than that of the younger patients (24.9 compared to 39.7 months). This difference was markedly reduced in the DSS, implicating that not only the breast cancer, but also other factors influence the survival of elderly patients. Indeed, a Cox proportional hazards model showed that not only higher age at diagnosis, but also comorbidities, a higher number of metastatic sites, high tumour grading or negative HR- or HER2-status increased the risk of mortality. These results are in concordance with the literature about prognostic factors in metastatic breast cancer [7, 31–33]. Interestingly, obesity (BMI ≥ 30) was associated with a decreased risk of mortality, the opposite of what was shown previously [34]. Synchronous metastasis at diagnosis was also favourable, a fact that could be attributed to the missing previous adjuvant chemotherapy, which has been identified as predicting factor negatively influencing survival [7, 35, 36]. Thus, the assessment of treatment and outcome of elderly patients should consider that factors other than age also affect decision making and prognosis.

## Conclusions

The results from the SENORA project add valuable information to the ongoing debate about ‘undertreatment’ of elderly patients—there was no substantial difference in the treatment of elderly women with breast cancer in German routine care. Age was but one of various prognostic factors influencing the survival of elderly patients and—despite similar treatment—the overall survival of patients aged ≥ 70 was shorter.
